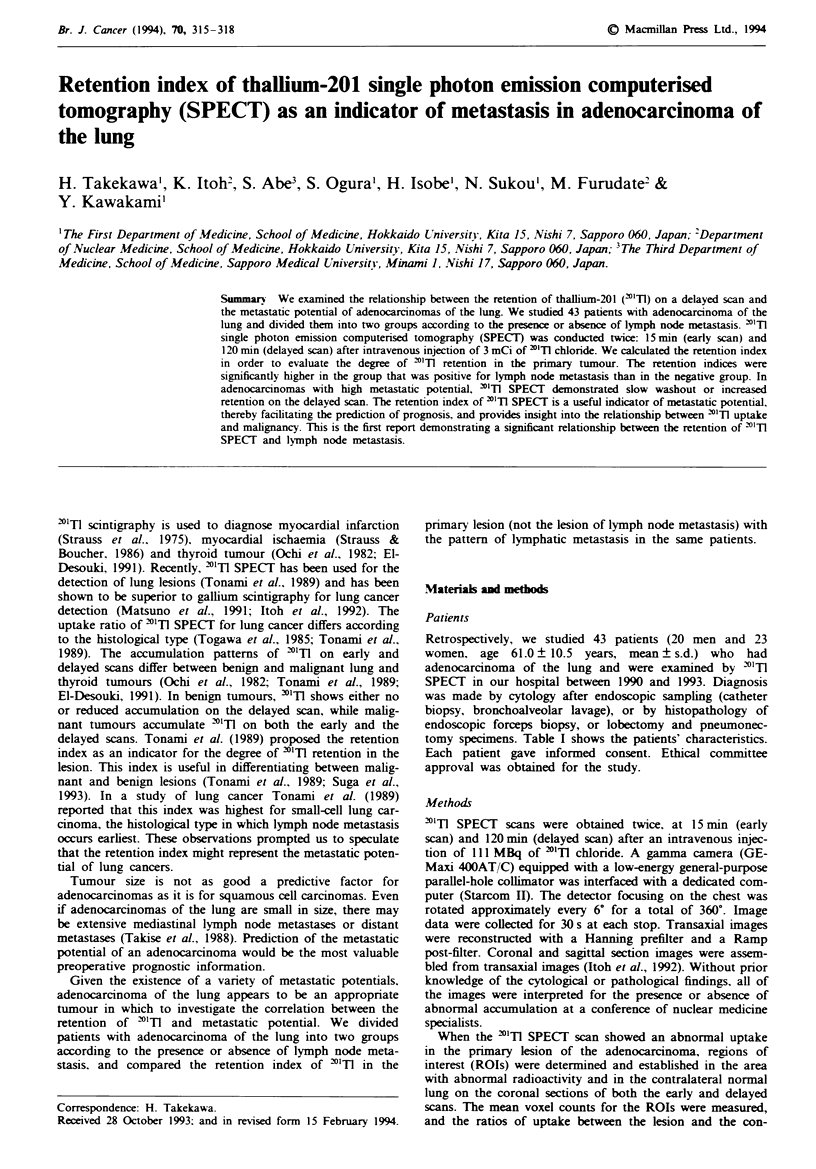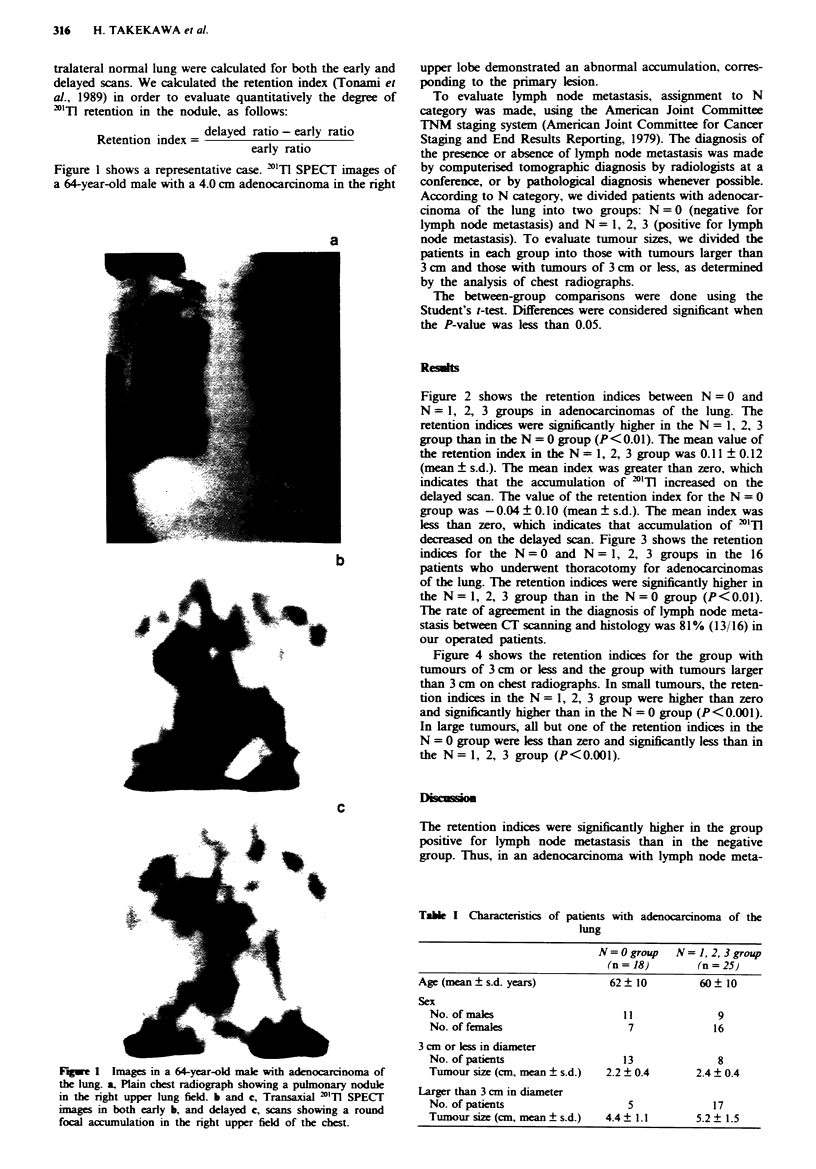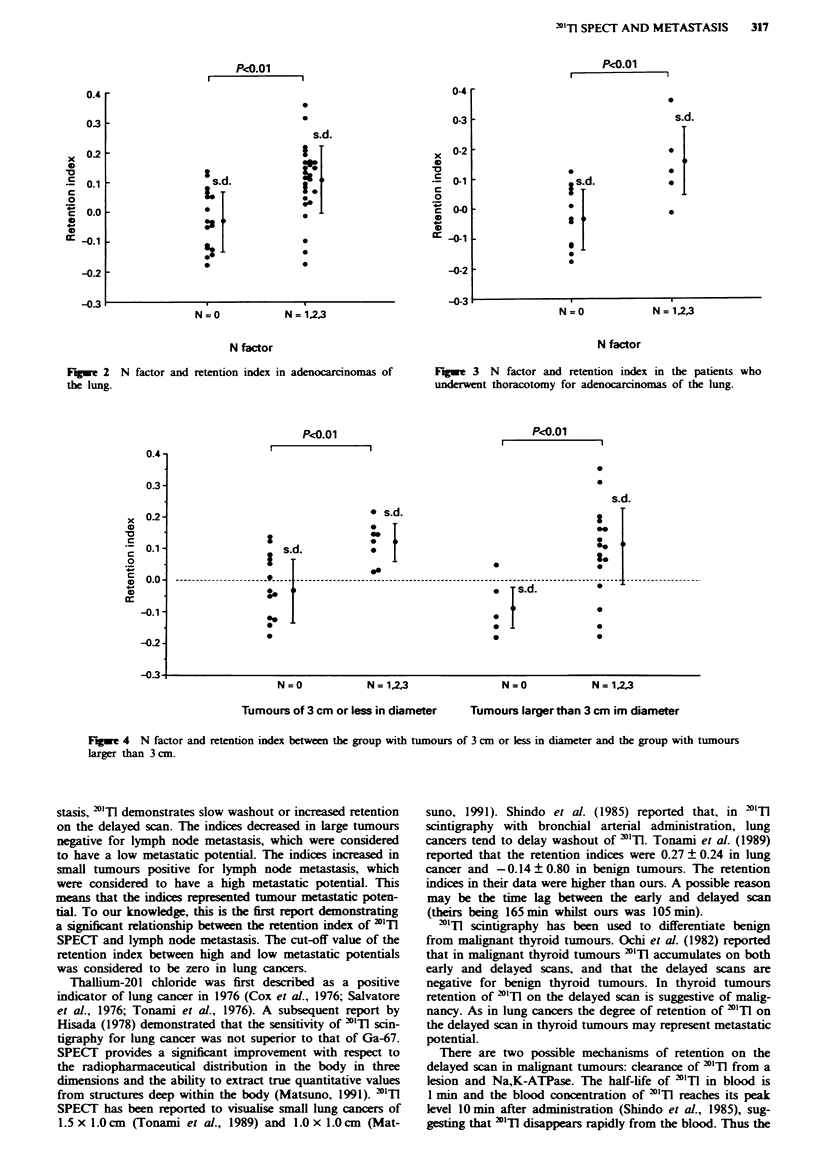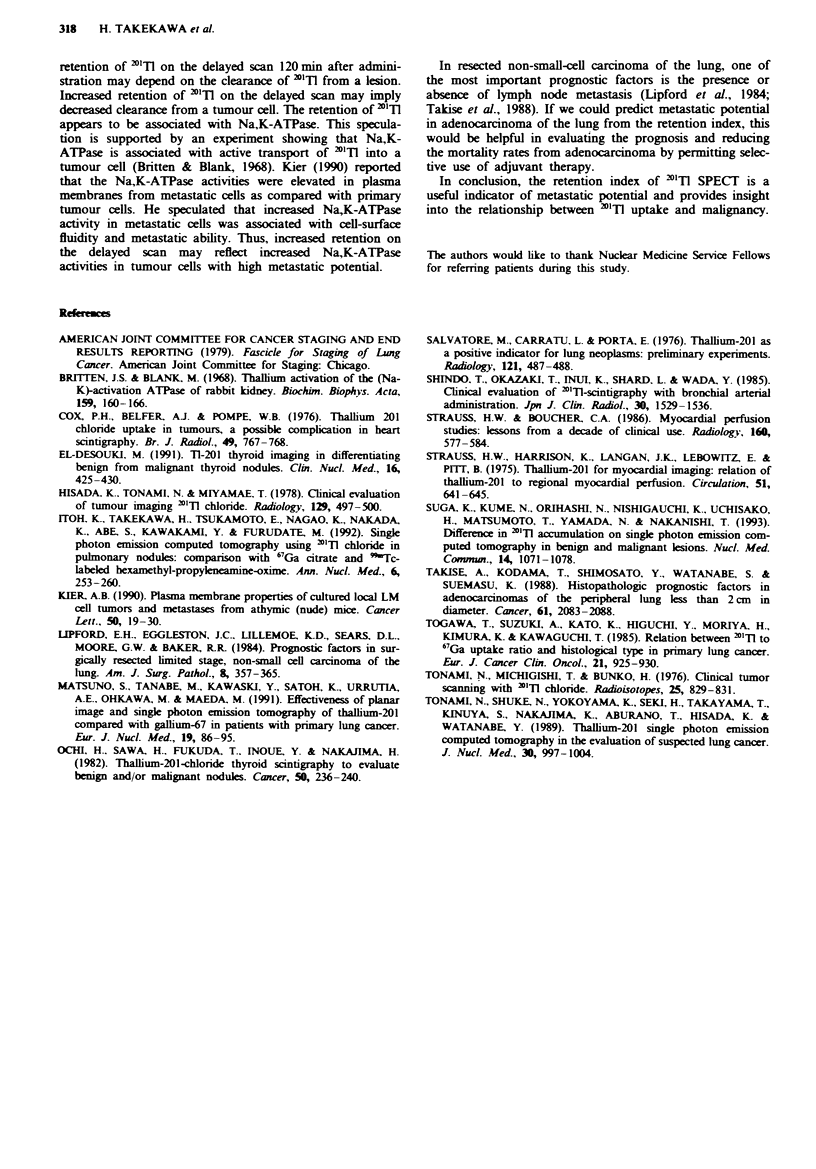# Retention index of thallium-201 single photon emission computerised tomography (SPECT) as an indicator of metastasis in adenocarcinoma of the lung.

**DOI:** 10.1038/bjc.1994.299

**Published:** 1994-08

**Authors:** H. Takekawa, K. Itoh, S. Abe, S. Ogura, H. Isobe, N. Sukou, M. Furudate, Y. Kawakami

**Affiliations:** First Department of Medicine, School of Medicine, Hokkaido University, Sapporo, Japan.

## Abstract

**Images:**


Br. J. Cancer (1994), 70, 315-318 © Macmillan Press Ltd., 1994

### Retention index of thallium-201 single photon emission computerised tomography (SPECT) as an indicator of metastasis in adenocarcinoma of the lung

H. Takekawa[1], K. Itoh[2], S. Abe[3], S. Ogura[1], H. Isobe[1], N. Sukou[1], M. Furudate[2] & Y. Kawakami[1]

[1]*The First Department of Medicine, School of Medicine, Hokkaido University, Kita 15, Nishi 7, Sapporo 060, Japan;* [2]*Department of Nuclear Medicine, School of Medicine, Hokkaido University, Kita 15, Nishi 7, Sapporo 060, Japan;* [3]*The Third Department of Medicine, School of Medicine, Sapporo Medical University, Minami 1, Nishi 17, Sapporo 060, Japan.*

**Summary** We examined the relationship between the retention of thallium-201 ($^{201}$Tl) on a delayed scan and the metastatic potential of adenocarcinomas of the lung. We studied 43 patients with adenocarcinoma of the lung and divided them into two groups according to the presence or absence of lymph node metastasis. $^{201}$Tl single photon emission computerised tomography (SPECT) was conducted twice: 15 min (early scan) and 120 min (delayed scan) after intravenous injection of 3 mCi of $^{201}$Tl chloride. We calculated the retention index in order to evaluate the degree of $^{201}$Tl retention in the primary tumour. The retention indices were significantly higher in the group that was positive for lymph node metastasis than in the negative group. In adenocarcinomas with high metastatic potential, $^{201}$Tl SPECT demonstrated slow washout or increased retention on the delayed scan. The retention index of $^{201}$Tl SPECT is a useful indicator of metastatic potential, thereby facilitating the prediction of prognosis, and provides insight into the relationship between $^{201}$Tl uptake and malignancy. This is the first report demonstrating a significant relationship between the retention of $^{201}$Tl SPECT and lymph node metastasis.

$^{201}$Tl scintigraphy is used to diagnose myocardial infarction (Strauss *et al.*, 1975), myocardial ischaemia (Strauss & Boucher, 1986) and thyroid tumour (Ochi *et al.*, 1982; El-Desouki, 1991). Recently, $^{201}$Tl SPECT has been used for the detection of lung lesions (Tonami *et al.*, 1989) and has been shown to be superior to gallium scintigraphy for lung cancer detection (Matsuno *et al.*, 1991; Itoh *et al.*, 1992). The uptake ratio of $^{201}$Tl SPECT for lung cancer differs according to the histological type (Togawa *et al.*, 1985; Tonami *et al.*, 1989). The accumulation patterns of $^{201}$Tl on early and delayed scans differ between benign and malignant lung and thyroid tumours (Ochi *et al.*, 1982; Tonami *et al.*, 1989; El-Desouki, 1991). In benign tumours, $^{201}$Tl shows either no or reduced accumulation on the delayed scan, while malignant tumours accumulate $^{201}$Tl on both the early and the delayed scans. Tonami *et al.* (1989) proposed the retention index as an indicator for the degree of $^{201}$Tl retention in the lesion. This index is useful in differentiating between malignant and benign lesions (Tonami *et al.*, 1989; Suga *et al.*, 1993). In a study of lung cancer Tonami *et al.* (1989) reported that this index was highest for small-cell lung carcinoma, the histological type in which lymph node metastasis occurs earliest. These observations prompted us to speculate that the retention index might represent the metastatic potential of lung cancers.

Tumour size is not as good a predictive factor for adenocarcinomas as it is for squamous cell carcinomas. Even if adenocarcinomas of the lung are small in size, there may be extensive mediastinal lymph node metastases or distant metastases (Takise *et al.*, 1988). Prediction of the metastatic potential of an adenocarcinoma would be the most valuable preoperative prognostic information.

Given the existence of a variety of metastatic potentials, adenocarcinoma of the lung appears to be an appropriate tumour in which to investigate the correlation between the retention of $^{201}$Tl and metastatic potential. We divided patients with adenocarcinoma of the lung into two groups according to the presence or absence of lymph node metastasis, and compared the retention index of $^{201}$Tl in the primary lesion (not the lesion of lymph node metastasis) with the pattern of lymphatic metastasis in the same patients.

Correspondence: H. Takekawa.
Received 28 October 1993; and in revised form 15 February 1994.

### Materials and methods

### *Patients*

Retrospectively, we studied 43 patients (20 men and 23 women, age 61.0 ± 10.5 years, mean ± s.d.) who had adenocarcinoma of the lung and were examined by $^{201}$Tl SPECT in our hospital between 1990 and 1993. Diagnosis was made by cytology after endoscopic sampling (catheter biopsy, bronchoalveolar lavage), or by histopathology of endoscopic forceps biopsy, or lobectomy and pneumonectomy specimens. Table I shows the patients' characteristics. Each patient gave informed consent. Ethical committee approval was obtained for the study.

### *Methods*

$^{201}$Tl SPECT scans were obtained twice, at 15 min (early scan) and 120 min (delayed scan) after an intravenous injection of 111 MBq of $^{201}$Tl chloride. A gamma camera (GE-Maxi 400AT/C) equipped with a low-energy general-purpose parallel-hole collimator was interfaced with a dedicated computer (Starcom II). The detector focusing on the chest was rotated approximately every 6° for a total of 360°. Image data were collected for 30 s at each stop. Transaxial images were reconstructed with a Hanning prefilter and a Ramp post-filter. Coronal and sagittal section images were assembled from transaxial images (Itoh *et al.*, 1992). Without prior knowledge of the cytological or pathological findings, all of the images were interpreted for the presence or absence of abnormal accumulation at a conference of nuclear medicine specialists.

When the $^{201}$Tl SPECT scan showed an abnormal uptake in the primary lesion of the adenocarcinoma, regions of interest (ROIs) were determined and established in the area with abnormal radioactivity and in the contralateral normal lung on the coronal sections of both the early and delayed scans. The mean voxel counts for the ROIs were measured, and the ratios of uptake between the lesion and the contralateral normal lung were calculated for both the early and delayed scans. We calculated the retention index (Tonami et al., 1989) in order to evaluate quantitatively the degree of $^{201}$Tl retention in the nodule, as follows:

$$\text{Retention index} = \frac{\text{delayed ratio} - \text{early ratio}}{\text{early ratio}}$$

Figure 1 shows a representative case. $^{201}$Tl SPECT images of a 64-year-old male with a 4.0 cm adenocarcinoma in the right upper lobe demonstrated an abnormal accumulation, corresponding to the primary lesion.

**Figure 1** Images in a 64-year-old male with adenocarcinoma of the lung. **a**, Plain chest radiograph showing a pulmonary nodule in the right upper lung field. **b** and **c**, Transaxial $^{201}$Tl SPECT images in both early **b**, and delayed **c**, scans showing a round focal accumulation in the right upper field of the chest.

To evaluate lymph node metastasis, assignment to N category was made, using the American Joint Committee TNM staging system (American Joint Committee for Cancer Staging and End Results Reporting, 1979). The diagnosis of the presence or absence of lymph node metastasis was made by computerised tomographic diagnosis by radiologists at a conference, or by pathological diagnosis whenever possible. According to N category, we divided patients with adenocarcinoma of the lung into two groups: N = 0 (negative for lymph node metastasis) and N = 1, 2, 3 (positive for lymph node metastasis). To evaluate tumour sizes, we divided the patients in each group into those with tumours larger than 3 cm and those with tumours of 3 cm or less, as determined by the analysis of chest radiographs.

The between-group comparisons were done using the Student's $t$-test. Differences were considered significant when the $P$-value was less than 0.05.

### Results

Figure 2 shows the retention indices between N = 0 and N = 1, 2, 3 groups in adenocarcinomas of the lung. The retention indices were significantly higher in the N = 1, 2, 3 group than in the N = 0 group ($P<0.01$). The mean value of the retention index in the N = 1, 2, 3 group was 0.11 ± 0.12 (mean ± s.d.). The mean index was greater than zero, which indicates that the accumulation of $^{201}$Tl increased on the delayed scan. The value of the retention index for the N = 0 group was −0.04 ± 0.10 (mean ± s.d.). The mean index was less than zero, which indicates that accumulation of $^{201}$Tl decreased on the delayed scan. Figure 3 shows the retention indices for the N = 0 and N = 1, 2, 3 groups in the 16 patients who underwent thoracotomy for adenocarcinomas of the lung. The retention indices were significantly higher in the N = 1, 2, 3 group than in the N = 0 group ($P<0.01$). The rate of agreement in the diagnosis of lymph node metastasis between CT scanning and histology was 81% (13/16) in our operated patients.

Figure 4 shows the retention indices for the group with tumours of 3 cm or less and the group with tumours larger than 3 cm on chest radiographs. In small tumours, the retention indices in the N = 1, 2, 3 group were higher than zero and significantly higher than in the N = 0 group ($P<0.001$). In large tumours, all but one of the retention indices in the N = 0 group were less than zero and significantly less than in the N = 1, 2, 3 group ($P<0.001$).

### Discussion

The retention indices were significantly higher in the group positive for lymph node metastasis than in the negative group. Thus, in an adenocarcinoma with lymph node meta-

**Table I** Characteristics of patients with adenocarcinoma of the lung

| | *N = 0 group* (n = *18*) | *N = 1, 2, 3 group* (n = *25*) |
|---|---|---|
| Age (mean ± s.d. years) | 62 ± 10 | 60 ± 10 |
| Sex | | |
| No. of males | 11 | 9 |
| No. of females | 7 | 16 |
| 3 cm or less in diameter | | |
| No. of patients | 13 | 8 |
| Tumour size (cm, mean ± s.d.) | 2.2 ± 0.4 | 2.4 ± 0.4 |
| Larger than 3 cm in diameter | | |
| No. of patients | 5 | 17 |
| Tumour size (cm, mean ± s.d.) | 4.4 ± 1.1 | 5.2 ± 1.5 |

Figure 2 N factor and retention index in adenocarcinomas of the lung.

Figure 3 N factor and retention index in the patients who underwent thoracotomy for adenocarcinomas of the lung.

Figure 4 N factor and retention index between the group with tumours of 3 cm or less in diameter and the group with tumours larger than 3 cm.

stasis, $^{201}$Tl demonstrates slow washout or increased retention on the delayed scan. The indices decreased in large tumours negative for lymph node metastasis, which were considered to have a low metastatic potential. The indices increased in small tumours positive for lymph node metastasis, which were considered to have a high metastatic potential. This means that the indices represented tumour metastatic potential. To our knowledge, this is the first report demonstrating a significant relationship between the retention index of $^{201}$Tl SPECT and lymph node metastasis. The cut-off value of the retention index between high and low metastatic potentials was considered to be zero in lung cancers.

Thallium-201 chloride was first described as a positive indicator of lung cancer in 1976 (Cox *et al.*, 1976; Salvatore *et al.*, 1976; Tonami *et al.*, 1976). A subsequent report by Hisada (1978) demonstrated that the sensitivity of $^{201}$Tl scintigraphy for lung cancer was not superior to that of Ga-67. SPECT provides a significant improvement with respect to the radiopharmaceutical distribution in the body in three dimensions and the ability to extract true quantitative values from structures deep within the body (Matsuno, 1991). $^{201}$Tl SPECT has been reported to visualise small lung cancers of 1.5 × 1.0 cm (Tonami *et al.*, 1989) and 1.0 × 1.0 cm (Matsuno, 1991). Shindo *et al.* (1985) reported that, in $^{201}$Tl scintigraphy with bronchial arterial administration, lung cancers tend to delay washout of $^{201}$Tl. Tonami *et al.* (1989) reported that the retention indices were $0.27 \pm 0.24$ in lung cancer and $-0.14 \pm 0.80$ in benign tumours. The retention indices in their data were higher than ours. A possible reason may be the time lag between the early and delayed scan (theirs being 165 min whilst ours was 105 min).

$^{201}$Tl scintigraphy has been used to differentiate benign from malignant thyroid tumours. Ochi *et al.* (1982) reported that in malignant thyroid tumours $^{201}$Tl accumulates on both early and delayed scans, and that the delayed scans are negative for benign thyroid tumours. In thyroid tumours retention of $^{201}$Tl on the delayed scan is suggestive of malignancy. As in lung cancers the degree of retention of $^{201}$Tl on the delayed scan in thyroid tumours may represent metastatic potential.

There are two possible mechanisms of retention on the delayed scan in malignant tumours: clearance of $^{201}$Tl from a lesion and Na,K-ATPase. The half-life of $^{201}$Tl in blood is 1 min and the blood concentration of $^{201}$Tl reaches its peak level 10 min after administration (Shindo *et al.*, 1985), suggesting that $^{201}$Tl disappears rapidly from the blood. Thus the retention of $^{201}Tl$ on the delayed scan 120 min after administration may depend on the clearance of $^{201}Tl$ from a lesion. Increased retention of $^{201}Tl$ on the delayed scan may imply decreased clearance from a tumour cell. The retention of $^{201}Tl$ appears to be associated with Na,K-ATPase. This speculation is supported by an experiment showing that Na,K-ATPase is associated with active transport of $^{201}Tl$ into a tumour cell (Britten & Blank, 1968). Kier (1990) reported that the Na,K-ATPase activities were elevated in plasma membranes from metastatic cells as compared with primary tumour cells. He speculated that increased Na,K-ATPase activity in metastatic cells was associated with cell-surface fluidity and metastatic ability. Thus, increased retention on the delayed scan may reflect increased Na,K-ATPase activities in tumour cells with high metastatic potential.

In resected non-small-cell carcinoma of the lung, one of the most important prognostic factors is the presence or absence of lymph node metastasis (Lipford *et al.*, 1984; Takise *et al.*, 1988). If we could predict metastatic potential in adenocarcinoma of the lung from the retention index, this would be helpful in evaluating the prognosis and reducing the mortality rates from adenocarcinoma by permitting selective use of adjuvant therapy.

In conclusion, the retention index of $^{201}Tl$ SPECT is a useful indicator of metastatic potential and provides insight into the relationship between $^{201}Tl$ uptake and malignancy.

The authors would like to thank Nuclear Medicine Service Fellows for referring patients during this study.

**References**

AMERICAN JOINT COMMITTEE FOR CANCER STAGING AND END RESULTS REPORTING (1979). *Fascicle for Staging of Lung Cancer.* American Joint Committee for Staging: Chicago.

BRITTEN, J.S. & BLANK, M. (1968). Thallium activation of the (Na-K)-activation ATPase of rabbit kidney. *Biochim. Biophys. Acta*, **159**, 160–166.

COX, P.H., BELFER, A.J. & POMPE, W.B. (1976). Thallium 201 chloride uptake in tumours, a possible complication in heart scintigraphy. *Br. J. Radiol.*, **49**, 767–768.

EL-DESOUKI, M. (1991). Tl-201 thyroid imaging in differentiating benign from malignant thyroid nodules. *Clin. Nucl. Med.*, **16**, 425–430.

HISADA, K., TONAMI, N. & MIYAMAE, T. (1978). Clinical evaluation of tumour imaging $^{201}Tl$ chloride. *Radiology*, **129**, 497–500.

ITOH, K., TAKEKAWA, H., TSUKAMOTO, E., NAGAO, K., NAKADA, K., ABE, S., KAWAKAMI, Y. & FURUDATE, M. (1992). Single photon emission computed tomography using $^{201}Tl$ chloride in pulmonary nodules: comparison with $^{67}Ga$ citrate and $^{99m}Tc$-labeled hexamethyl-propyleneamine-oxime. *Ann. Nucl. Med.*, **6**, 253–260.

KIER, A.B. (1990). Plasma membrane properties of cultured local LM cell tumors and metastases from athymic (nude) mice. *Cancer Lett.*, **50**, 19–30.

LIPFORD, E.H., EGGLESTON, J.C., LILLEMOE, K.D., SEARS, D.L., MOORE, G.W. & BAKER, R.R. (1984). Prognostic factors in surgically resected limited stage, non-small cell carcinoma of the lung. *Am. J. Surg. Pathol.*, **8**, 357–365.

MATSUNO, S., TANABE, M., KAWASKI, Y., SATOH, K., URRUTIA, A.E., OHKAWA, M. & MAEDA, M. (1991). Effectiveness of planar image and single photon emission tomography of thallium-201 compared with gallium-67 in patients with primary lung cancer. *Eur. J. Nucl. Med.*, **19**, 86–95.

OCHI, H., SAWA, H., FUKUDA, T., INOUE, Y. & NAKAJIMA, H. (1982). Thallium-201-chloride thyroid scintigraphy to evaluate benign and/or malignant nodules. *Cancer*, **50**, 236–240.

SALVATORE, M., CARRATU, L. & PORTA, E. (1976). Thallium-201 as a positive indicator for lung neoplasms: preliminary experiments. *Radiology*, **121**, 487–488.

SHINDO, T., OKAZAKI, T., INUI, K., SHARD, L. & WADA, Y. (1985). Clinical evaluation of $^{201}Tl$-scintigraphy with bronchial arterial administration. *Jpn J. Clin. Radiol.*, **30**, 1529–1536.

STRAUSS, H.W. & BOUCHER, C.A. (1986). Myocardial perfusion studies: lessons from a decade of clinical use. *Radiology*, **160**, 577–584.

STRAUSS, H.W., HARRISON, K., LANGAN, J.K., LEBOWITZ, E. & PITT, B. (1975). Thallium-201 for myocardial imaging: relation of thallium-201 to regional myocardial perfusion. *Circulation*, **51**, 641–645.

SUGA, K., KUME, N., ORIHASHI, N., NISHIGAUCHI, K., UCHISAKO, H., MATSUMOTO, T., YAMADA, N. & NAKANISHI, T. (1993). Difference in $^{201}Tl$ accumulation on single photon emission computed tomography in benign and malignant lesions. *Nucl. Med. Commun.*, **14**, 1071–1078.

TAKISE, A., KODAMA, T., SHIMOSATO, Y., WATANABE, S. & SUEMASU, K. (1988). Histopathologic prognostic factors in adenocarcinomas of the peripheral lung less than 2 cm in diameter. *Cancer*, **61**, 2083–2088.

TOGAWA, T., SUZUKI, A., KATO, K., HIGUCHI, Y., MORIYA, H., KIMURA, K. & KAWAGUCHI, T. (1985). Relation between $^{201}Tl$ to $^{67}Ga$ uptake ratio and histological type in primary lung cancer. *Eur. J. Cancer Clin. Oncol.*, **21**, 925–930.

TONAMI, N., MICHIGISHI, T. & BUNKO, H. (1976). Clinical tumor scanning with $^{201}Tl$ chloride. *Radioisotopes*, **25**, 829–831.

TONAMI, N., SHUKE, N., YOKOYAMA, K., SEKI, H., TAKAYAMA, T., KINUYA, S., NAKAJIMA, K., ABURANO, T., HISADA, K. & WATANABE, Y. (1989). Thallium-201 single photon emission computed tomography in the evaluation of suspected lung cancer. *J. Nucl. Med.*, **30**, 997–1004.